# Nursing Progress and Its Developments: IV. Endowments for the College

**Published:** 1918-01-05

**Authors:** 


### THE MATRONS' AND SISTERS' DEPARTMENT.

### NURSING PROGRESS AND ITS DEVELOPMENTS.

### IV. Endowments for the College.

FLORENCE NIGHTINGALE's views about the higher development of nursing and the scope it affords for women of brains and capacity are well known. She forms the best possible illustration in her own person of the value of brains to those whose energies are turned in this direction. All who have studied the many intricate national problems involved in the vocation of nursing have reached the conclusion that they are such as to demand, from some at least who embrace it, years of continuous study. This is indeed a counsel of perfection unattainable by the women best qualified to devote themselves to such tasks unless the public make it possible by endowing scholarships for them. But short of such advanced students, every matron knows of cases where able nurses, who could easily have made their mark in administrative appointments, have been forced by poverty to give themselves to the comparatively shallow waters of private nursing. Many a nurse goes maimed, professionally speaking, to this day because she lacked the means to take the one additional course which would have doubled her market value. What nurses do not realise is that the College of Nursing has already set itself to open up opportunities, from which they have long been debarred, for women entering upon a nursing career.

The endowment of the College of Nursing is but the first step towards the realisation of aims which have hitherto been reckoned as lying outside practical politics. Isolated instances can be quoted of bequests and benefactions to particular institutions made for the assistance of probationers, but these have usually stopped short at the provision of prizes or medals to distinguish those who do well during their three years' course. What is imperatively needed, however, is a ladder which shall reach up above the standard curriculum and place a graduate course within the grasp of women who are capable of using it. Scholarships, limited in their scope to no particular training-school, can be made available for nurses solely through a central organisation, and the British Women's Hospital Committee in making its appeal for a National Gift to Nurses has kept the need for these scholarships steadily in view from the outset.

There may be some (we do not think there are many) nurses who hold this ideal cheaply. They can see the use of helping infirm nurses, but fail to realise why the young and the clever should have their path to distinction made easier. They are strangely mistaken in believing that the interests of the rank and file are dissociated from the more able members of the profession, for every rank includes some of the latter type. What we all earnestly desire for the nursing profession is to see it recruited from the best stock which the nation contains. Matrons are wont to deplore that the probationers of to-day are inferior in quality to those of their own youth, and though this complaint is often founded on but hazy recollections of the past, it is certain that there is far more competition now than there used to be for probationers likely to make their mark. Unless the best women are continuously attracted to nursing it must steadily deteriorate as a profession. But the best women are more often poor than not. They want some ground of confidence that they can ultimately obtain posts in which their talents will have scope. To attract them as students the training-schools must strengthen their curriculum. When they have won their certificates they must be set to travel and investigate. It must be proved to them that probationership is the portal to a career of eager, purposeful, invaluable work for the raising of suffering humanity. Thus alone can we win the women we want as recruits for the nursing army in its successful fight against disease and misery.

